# Molecular and Cellular Mechanisms of Synaptopathies

**DOI:** 10.1155/2017/2643943

**Published:** 2017-04-30

**Authors:** Alvaro O. Ardiles, Andreas M. Grabrucker, Francisco G. Scholl, Gabby Rudenko, Tiziana Borsello

**Affiliations:** ^1^Escuela de Medicina, Facultad de Medicina, Universidad de Valparaíso, 2341386 Valparaíso, Chile; ^2^Centro Interdisciplinario de Neurociencia de Valparaíso, Facultad de Ciencias, Universidad de Valparaíso, 2360102 Valparaíso, Chile; ^3^Institute for Anatomy and Cell Biology, Ulm University, 89081 Ulm, Germany; ^4^Department of Biological Sciences, University of Limerick, Limerick, Ireland; ^5^Instituto de Biomedicina de Sevilla (IBiS), Hospital Universitario Virgen del Rocío/CSIC/Universidad de Sevilla and Departamento de Fisiología Médica y Biofísica, Universidad de Sevilla, 41013 Sevilla, Spain; ^6^Department of Pharmacology and Toxicology, Sealy Center for Structural Biology and Molecular Biophysics, University of Texas Medical Branch, 301 University Boulevard Rm. 5.114B, Galveston, TX 77555, USA; ^7^IRCCS Istituto di Ricerche Farmacologiche “Mario Negri”, Via La Masa 19, 20156 Milan, Italy; ^8^Department of Pharmacological and Biomolecular Sciences, University of Milan, Milan, Italy

Synapses, contact points between neurons, are essential elements supporting the ability of neurons to communicate and to transmit relevant information to each other. They play an integral role in brain development and wiring neurons into neural circuits, for example, those related to our behavior. Therefore, alterations affecting the integrity and/or functionality of synapses can lead to synaptic pathologies or synaptopathies. For instance, many neurological disorders including Alzheimer's disease, Down syndrome, epilepsy, and Parkinson's disease and neurodevelopmental disorders such as autism spectrum disorders, intellectual disability, and fragile X syndrome have consistently been reported to exhibit abnormalities in synaptic composition, morphology, and function. This special issue discusses various aspects of the molecular interactions that underlie synaptic protein networks and the complex signaling pathways that are activated by them, knowledge that is crucial to understand the cellular and molecular mechanisms involved in different synaptopathies. Synapses comprise a presynaptic compartment, consisting of the axon terminal and their protein machinery implicated in the release of neurotransmitters. Upon exocytosis of presynaptic vesicles, neurotransmitters spill out into the extracellular space called the “synaptic cleft” and diffuse to reach a postsynaptic compartment, composed of the protein machinery that receives and transduces the neurotransmitter-induced signals [1]. Most synaptopathies directly or indirectly affect the molecular repertoire of synaptic proteins.

V. I. Torres et al. provide a comprehensive review describing specific pre- and postsynaptic proteins that are involved in the physiopathology of various synaptopathies and how deficits in these molecules contribute to different synaptopathic mechanisms. V. I. Torres et al. catalogue the different presynaptic and postsynaptic proteins that to date have been implicated in neuropsychiatric, neurodevelopmental, and neurodegenerative disorders. A more specific review by G. Rudenko focusses on synaptic adhesion molecules (SAMs), many of which are now implicated in neuropsychiatric, neurodevelopmental, and neurodegenerative diseases as well. SAMs tether to the pre- or postsynaptic membranes extending their extracellular domains into the synaptic cleft where they coordinate protein interaction networks. A much larger diversity of SAMs and their protein interactions exists than has previously been appreciated. In addition, SAMs drive more complex functions than purely the adhesion of presynaptic and postsynaptic membranes. Furthermore, SAMs are under dynamic control through a variety of mechanisms, enabling them to play a key role in plasticity at synapses. Thus, considering the crucial role that SAMs play in synapse development, they may yield novel therapeutic targets which can be exploited to ameliorate certain synaptopathies.

Several articles in this special issue focus on specific synaptic proteins in detail. S. Biggi et al. present evidence that c-Jun N-terminal kinase (JNK), acting presynaptically, may have an important functional role in synapses. JNK is part of a signaling pathway strongly activated by NMDA stimulation and involved in synaptic plasticity. It is noteworthy that until now, most studies have been focused on the postsynaptic mechanism of JNK action, and less is known about JNK presynaptic localization and its physiological role at this site. S. Biggi et al. demonstrate that activation of presynaptic NMDA receptors leads to the activation of JNK which modulates neurotransmitter release through a direct interaction with SNARE proteins. These findings are relevant considering that JNK activity had been associated with not only neurodegenerative disorders like Alzheimer's, Huntington's, and Parkinson's disease but also psychiatric disorders and intellectual disabilities. F. Longhena et al. review recent evidence indicating that *α*-synuclein possesses a novel “prion-like” behavior, whereby the protein can spread transsynaptically to trigger the aggregation of *α*-synuclein in neighboring neurons, a phenomenon observed in Parkinson's disease animal models and patients [2–5]. Monomeric, oligomeric, and fibrillary *α*-synuclein forms accumulate at the synaptic terminal due to their high affinity for vesicular membranes and can be released in a process that is thought to be mediated mainly by exosomal vesicles [6, 7]. Once released, *α*-synuclein could affect endogenous *α*-synuclein on recipient neurons, thereby affecting their function, and/or could interact with membrane lipids leading to both pre- and postsynaptic alterations and hence synaptic failure. The elucidation of this novel mechanism of *α*-synuclein transmission can shed lights on the contribution of *α*-synuclein spreading to Parkinson's disease related synaptopathy. E. Pérez-Palma et al. introduce new evidence relating to Wnt/*β*-catenin signaling and the expression of novel Wnt/*β*-catenin target genes. In addition to genes involved in neural precursors, forebrain development, and stem cell differentiation, E. Pérez-Palma et al. also identified a significant number of genes with transcription factor activity. The genes modulated by Wnt/*β*-catenin signaling are involved in biological processes such as neuronal structure and activity, and that are affected in synaptopathies. B. K. Unda et al. provide evidence that the secreted neurotrophic factor neuregulin-1 (NRG1) and its receptor ErbB4 signal through the multifunctional scaffold protein, disrupted in schizophrenia 1 (DISC1). Together, they regulate the development of cortical inhibitory interneurons and thus also impact the proper balance between excitatory and inhibitory transmissions. Since the genes encoding these proteins have been identified independently as risk factors for schizophrenia, a complete understanding of how these proteins interact to regulate the development of cortical inhibitory neuron morphology and synapse formation may provide insights into how these processes progress into synaptopathies.

Further, in this special issue, two papers focus on the use of animal models to yield a wealth of information about the underlying mechanisms of synaptopathies. Epilepsy is a neuropsychiatric condition characterized by an abnormal and excessive neural activity leading to a predisposition to recurrent unprovoked seizures affecting both excitatory and inhibitory synapses, synaptic plasticity, and behavior [8]. M. Lenz et al. use a pilocarpine-induced status epilepticus animal model to study synaptopodin, an actin-binding protein expressed in cortical neurons that is involved in the modulation of synaptic plasticity and spine remodeling [9, 10]. They propose that synaptopodin is a valuable diagnostic marker to detect alterations in the ability of neurons to undergo synaptic plasticity such as can be observed in seizure-induced synaptopathies; M. Lenz et al. provide further support for the role of synaptopodin in the ability of hippocampal neurons to exhibit synaptic plasticity, demonstrating that intraperitoneal pilocarpine injection reduces synaptopodin levels and affects the induction of long-term potentiation (LTP). M. I. Herrera et al. review the literature linking perinatal asphyxia and synaptic dysfunction obtained from an experimental model of perinatal asphyxia. Perinatal asphyxia is an obstetric complication resulting to abnormal brain development in term and preterm neonates; if affected babies survive, they develop neurological disorders such as epilepsy, cerebral palsy, mental retardation, attention-deficit disorder, and schizophrenia. M. I. Herrera et al. present evidence that perinatal asphyxia induces a series of modifications in synaptic composition, structure, and function leading to alterations in synapses and their function.

Lastly, focusing on the role of gene-environment interaction in mental disorders, S. Pfaender et al. provide evidence that zinc signaling plays a role in proliferation and neuronal differentiation of stem cells. They show that zinc deficiency during brain development impairs neurogenesis and modulates the expression of synaptic proteins, primary mechanisms influencing brain function.

In summary, articles published in this special issue highlight the very complex nature of synaptopathies, the participation of a very diverse portfolio of synaptic proteins linked to synaptopathies, and emphasize the importance of understanding these proteins to identify potential novel targets which might benefit the development of therapies for neuropsychiatric, neurodevelopment, and neurodegenerative diseases.
